# Functional-level analysis of post-stroke social participation: A nationwide cohort study using the ICF framework

**DOI:** 10.1371/journal.pone.0354691

**Published:** 2026-07-23

**Authors:** Jinhee Choi, Junhee Han, Hae In Lee, Min Kyun Sohn, Jongmin Lee, Deog Young Kim, Gyung Jae Oh, Yang-Soo Lee, Min Cheol Joo, So Young Lee, Min-Keun Song, Jeonghoon Ahn, Yun-Hee Kim, Ji Hong Min, Sung-Hwa Ko, Won Hyuk Chang, Yong-Il Shin

**Affiliations:** 1 Department of Rehabilitation Medicine, Pusan National University School of Medicine, Pusan National University Yangsan Hospital, Yangsan, Republic of Korea; 2 Department of Statistics, Hallym University, Chuncheon, Republic of Korea; 3 Chronic Disease Control Division, Gyeongnam Regional Center for Disease Control and Prevention, Busan, Republic of Korea; 4 Department of Rehabilitation Medicine, School of Medicine, Chungnam National University, Daejeon, Republic of Korea; 5 Department of Rehabilitation Medicine, Konkuk University School of Medicine, Seoul, Republic of Korea; 6 Department and Research Institute of Rehabilitation Medicine, Yonsei University College of Medicine, Seoul, Republic of Korea; 7 Department of Preventive Medicine, Wonkwang University School of Medicine, Iksan, Republic of Korea; 8 Department of Rehabilitation Medicine, Kyungpook National University School of Medicine, Kyungpook National University Hospital, Daegu, Republic of Korea; 9 Department of Rehabilitation Medicine, Wonkwang University School of Medicine, Iksan, Republic of Korea; 10 Department of Rehabilitation Medicine, Jeju National University School of Medicine, Jeju, Republic of Korea; 11 Department of Physical and Rehabilitation Medicine, Chonnam National University Medical School, Gwangju, Republic of Korea; 12 Department of Health Convergence, Ewha Womans University, Seoul, Republic of Korea; 13 Department of Physical and Rehabilitation Medicine, Sungkyunkwan University School of Medicine, Suwon, Republic of Korea; 14 Research Institute for Convergence of Biomedical Science and Technology, Pusan National University Yangsan Hospital, Yangsan, Republic of Korea; 15 Department of Physical and Rehabilitation Medicine, Center for Prevention and Rehabilitation, Heart Vascular and Stroke Institute, Samsung Medical Center, Sungkyunkwan University School of Medicine, Seoul, Republic of Korea; 16 Department of Health Sciences and Technology, Department of Medical Device Management & Research, Department of Digital Health, SAIHST, Sungkyunkwan University, Seoul, Republic of Korea; PLOS: Public Library of Science, UNITED KINGDOM OF GREAT BRITAIN AND NORTHERN IRELAND

## Abstract

**Background:**

Social participation is a key goal in stroke rehabilitation, but its influencing factors vary by functional status. Few large-scale studies have categorized patients by functional level and analyzed associated factors using the International Classification of Functioning, Disability and Health (ICF) framework.

**Objective:**

To identify determinants of social participation 12 months after stroke, stratified by functional level.

**Design and participants:**

A cross-sectional analysis was conducted on 2,695 stroke survivors from a nationwide cohort in Korea. Participants were stratified into five groups based on Fugl-Meyer Assessment scores: Severe, Marked, Moderate, Slight, and Unimpaired.

**Methods:**

Social participation was measured using the Reintegration to Normal Living Index. Independent variables were categorized into four ICF domains: personal factors, body functions and structures, activities and participation, and environmental factors. Multivariate regression analyses were performed by functional group.

**Results:**

Predictors varied significantly across functional levels. In the Severe group (FMA < 50), environmental factors (home accessibility and caregiver-related variables such as age and relationship) were the primary determinants. Conversely, in higher-functioning groups (FMA ≥ 85), social participation was more strongly influenced by body functions and structures (cognitive, language, and psychosocial functions) and activities and participation factors (vocational status). Across all groups, activities and participation (functional independence and health-related quality of life) remained consistent positive predictors, while environmental barriers (caregiver burden) were universal constraints.

**Conclusion:**

Determinants of social participation differ by functional status: individuals with lower functional levels are more significantly affected by environmental factors, whereas those with higher function face greater barriers related to body functions and structures. These findings suggest that stroke rehabilitation must provide functional-level-specific interventions, moving beyond physical recovery to address these distinct multidimensional barriers.

## Introduction

Stroke remains one of the leading causes of long-term disability worldwide and imposes substantial physical, psychological, and social burdens on individuals, families, and healthcare systems. Although global stroke mortality has decreased in recent years due to advances in acute medical care, a substantial proportion of stroke survivors experience persistent disabilities, impairing their ability to live independently and engage in social activities [1,2]. Approximately 5 million people worldwide are left permanently disabled each year due to stroke [3]. As such, rehabilitation and social reintegration have become critical components of stroke recovery.

Many stroke survivors experience restrictions in activities of daily living (ADL) and limitations in social participation—even those with mild impairments [4,5]. Regaining the ability to participate in community and social life is widely recognized as a core goal of rehabilitation [6]. However, the focus of many stroke rehabilitation studies and clinical interventions has remained heavily skewed toward physical recovery alone [7].

The promotion of social participation after stroke requires a multidimensional approach. The International Classification of Functioning, Disability and Health (ICF) provides a comprehensive framework that considers not only body function but also activity, participation, and contextual (personal and environmental) factors [8].

In this study, social participation is defined as a person’s involvement in life situations, encompassing both the actual performance of activities and the subjective experience of that involvement [9]. This definition is operationalized through the Reintegration to Normal Living Index (RNLI), which specifically captures the survivor’s perception of their reintegration. Such a multidimensional approach is supported by Engel-Yeger et al. [10] who emphasize that participation is a complex construct and that recovery should be measured via patient-reported outcomes to reflect the true quality of life after stroke. Despite the clinical importance of these multidimensional factors, identifying the specific predictors of social participation requires large-scale empirical data to ensure statistical power and generalizability. Given that social participation is affected by the extent of impairment, categorizing patients by functional status enables more precise identification of influencing factors. Individuals with mild impairment may face cognitive or psychological barriers, while those with severe disabilities may be more affected by environmental and caregiving factors. Tailoring interventions according to functional level may improve rehabilitation outcomes and resource allocation.

While studies like Restore4Stroke(n = 106, conducted in the Netherlands) [11] have investigated social participation changes and START-PrePARE(n = 200, conducted in Australia) [12] have identified biological correlates of recovery, these investigations have been primarily limited to western populations with relatively small sample sizes. Our study builds on this prior research by addressing critical gaps in scale, population, and methodological innovation. First, in terms of scale, we utilize the nationwide KOSCO cohort, providing a robust sample of 2,695 survivors—a population size approximately 13–25 times larger than the aforementioned studies. Second, regarding population, this study focuses on Asian survivors in Korea, providing essential data from a region with unique sociocultural caregiving structures that differ from Western contexts. Third, we introduce a methodological innovation by employing functional stratification based on FMA thresholds, allowing for a more nuanced identification of predictors that are often masked in aggregate analyses.

By analyzing the large-scale KOSCO cohort, this study identifies distinct factors associated with 12-month post-stroke social participation across functional levels. These findings provide essential evidence for developing functionally stratified rehabilitation strategies to enhance social reintegration in Asian populations, where the burden of stroke remains significantly high [13].

## Methods

### *KOSCO* study

KOSCO is a 10-year longitudinal, multicenter, prospective cohort study of individuals with first-ever stroke admitted to participating hospitals across nine distinct regions in Korea. The rationale and protocols of the KOSCO study have been previously described [14]. This study was approved by the Research Ethics Committee of Pusan National University Yangsan Hospital (Institutional Review Board No. 05-2012-057) and the ethics committees of the other participating hospitals. All participants provided written informed consent prior to enrollment in the study. Only adult participants (aged 19 or older) were included.

### Selection and classification of participants

Data were collected from stroke patients enrolled during two separate recruitment periods in the KOSCO study: from August 1, 2012 to May 31, 2015 (first enrollment period), and from January 12, 2020 to December 31, 2020 (second enrollment period).

For all participants, follow-up assessments were conducted at 12 months after stroke onset, and the data from these 12-month follow-ups were analyzed in this study. The inclusion criteria were (a) first ever stroke with a corresponding lesion on magnetic resonance imaging/angiography, (b) age ≥ 19 years, (c) dwelling in the community, and (d) an understanding of the purpose of the study and provision of consent to participate. The exclusion criteria were (a) transient ischemic attack and (b) recurrent stroke. The integration of these two cohorts was justified as both recruitment waves utilized identical inclusion/exclusion criteria, assessment protocols, and standardized data collection forms according to the KOSCO guidelines. The second recruitment was conducted as an extension of the primary cohort study to increase the sample size and enhance the statistical power of the analysis. Furthermore, no significant demographic shifts or protocol-driven differences were observed between the periods, ensuring their comparability for aggregated analysis.

Participants who met the above criteria are again classified according to Fugl-Meyer Assessment (FMA) score as the Severe group (FMA < 50), the Marked group (50 ≤ FMA ≤ 84), the Moderate group (85 ≤ FMA ≤ 94), the Slight group (95 ≤ FMA ≤ 99) and the Unimpaired group (FMA = 100). These thresholds were selected based on the established clinical stages of motor recovery defined by Fugl-Meyer [15], which provide a standardized benchmark for post-stroke motor impairment severity.

### Data collection

People with stroke were first assessed within 7 days after stroke onset. Follow-up assessment was performed at 12 months, usually in the hospital setting. If the individuals were unable to visit the hospital, follow up home visits were conducted by trained occupational therapists at 12 months. All data were collected through both face to face interviews and according to the KOSCO guidelines. To ensure consistency and inter-rater reliability across the nine participating centers, all occupational therapists underwent standardized training according to the KOSCO study protocol before data collection. Periodic calibration sessions and monitoring were conducted throughout the study period to maintain high-quality data and minimize inter-assessor variability.

#### Dependent variables.

Social participation was measured using the Reintegration to Normal Living Index (RNLI), which was translated from the original RNLI [16] at 12 months of follow up and has demonstrated robust psychometric properties in stroke populations, including high internal consistency (Cronbach’s alpha > 0.90) and strong construct validity. This scale assesses how often stroke patients participate in 11 items, including involvement in mobility (indoor, community, distance), self care, daily activities, recreational activities, and social roles. Each item was scored from 0 to 10, with a maximum total score of 100 for the RNLI. Rather than measuring absolute frequency, the score reflects the degree of perceived reintegration; a score of 10 indicates that the patient has achieved a level of reintegration equivalent to their pre-stroke status or personal expectations, whereas a score of 0 represents a complete lack of reintegration in that domain. The total score was linearly transformed to a maximum of 100, where a higher RNLI score reflects a higher overall level of perceived social participation and successful reintegration.

#### Independent variables.

The 39 independent variables in this study were strategically extracted based on the biopsychosocial model of the ICF framework and the study protocols of prominent stroke cohorts, such as the Restore4Stroke [11] and START-PrePARE [12] studies, as well as data availability and clinical judgement. The rationale for selecting this comprehensive set of variables is that post-stroke social participation is a complex outcome determined by the dynamic interplay between an individual’s functional capacity and their contextual environment. Therefore, rather than focusing solely on physical recovery, we extracted a broad range of factors to capture the multifaceted barriers and facilitators to social integration.

**Fig 1:**
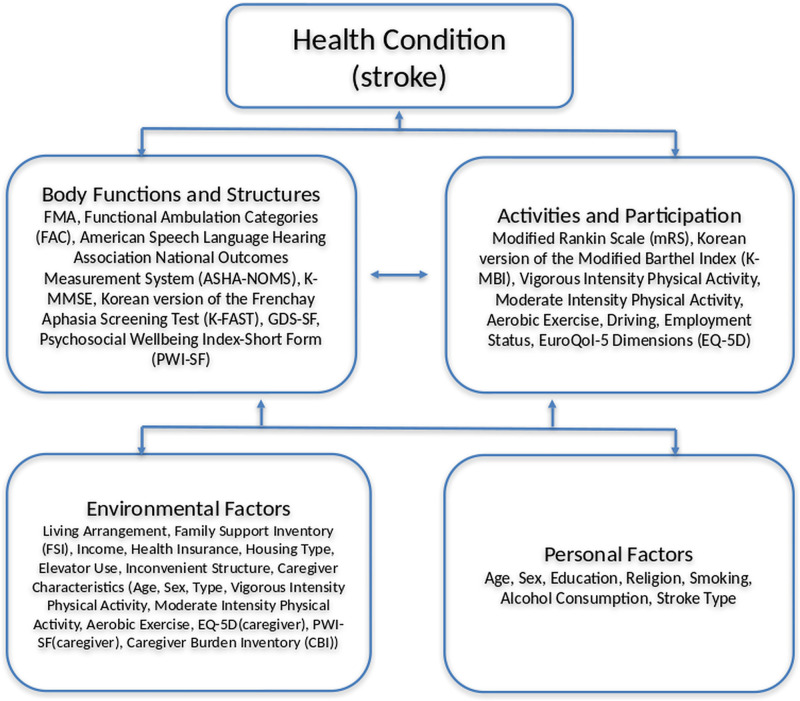
ICF-based classification of study variables.

All variables were systematically linked to the most appropriate ICF components following the refined linking rules proposed by Cieza et al [17]. Regarding the timing of data collection, all clinical independent variables used in the multivariate regression models were obtained at the 12-month follow-up assessment to ensure temporal alignment with the dependent variable (RNLI). Conversely, baseline demographics and initial clinical profiles (such as stroke type and baseline NIHSS) were recorded within 7 days of stroke onset. The variables were divided into four components, as follows: personal factors (7 variables) consisting of age, sex, education, religion, smoking, and alcohol consumption, stroke type; body functions and structures (7 variables) consisting of FMA [18], Functional Ambulation Categories (FAC) [19], American Speech Language Hearing Association National Outcomes Measurement System (ASHA-NOMS) [20], K-MMSE [21], Korean version of the Frenchay Aphasia Screening Test (K-FAST) [22], GDS-SF [23], and Psychosocial Wellbeing Index-Short Form (PWI-SF) [24]; activities and participation (8 variables) consisting of modified Rankin scale (mRS) [25], Korean version of the Modified Barthel Index (K-MBI) [26], vigorous intensity physical activity, moderate intensity physical activity, aerobic exercise, driving, employment status and EuroQol-5 dimensions (EQ-5D) [27] and environmental factors (16 variables) consisting of living arrangement, Family Support Inventory (FSI) [28], income, health insurance, housing type, elevator use, inconvenient structure, and caregiver characteristics (age, sex, type, vigorous intensity physical activity, moderate intensity physical activity, aerobic exercise, EQ-5D, PWI-SF, and Caregiver Burden Inventory (CBI) [29] (Fig 1, Table 1).

**Table 1:** The Classification of Variables According to ICF Components.

ICF Components	Variables
Personal Factors	Age, Sex, Education, Religion, Smoking, Alcohol Consumption, Stroke Type
Body Functions and Structures	FMA, Functional Ambulation Categories (FAC), American Speech Language Hearing Association National Outcomes Measurement System (ASHA-NOMS), K-MMSE, Korean version of the Frenchay Aphasia Screening Test (K-FAST), GDS-SF, Psychosocial Wellbeing Index-Short Form (PWI-SF)
Activities and Participation	Modified Rankin Scale (mRS), Korean version of the Modified Barthel Index (K-MBI), Vigorous Intensity Physical Activity, Moderate Intensity Physical Activity, Aerobic Exercise, Driving, Employment Status, EuroQol-5 Dimensions (EQ-5D)
Environmental Factors	Living Arrangement, Family Support Inventory (FSI), Income, Health Insurance, Housing Type, Elevator Use, Inconvenient Structure, Caregiver Characteristics (Age, Sex, Type, Vigorous Intensity Physical Activity, Moderate Intensity Physical Activity, Aerobic Exercise, EQ-5D, PWI-SF, Caregiver Burden Inventory (CBI))

The flowchart illustrates the 39 independent variables categorized into the four ICF domains: personal factors, body functions and structures, activities and participation, and environmental factors. Bidirectional arrows indicate the reciprocal interactions between components as defined by the WHO ICF framework.

#### Statistical analysis.

Numerical data are expressed as means and standard deviations (SDs), and categorical data are summarized as numbers and percentages. Statistical analyses were performed using IBM SPSS version 21.0 for Windows. The KOSCO dataset was accessed for research purposes between April 1, 2024 and May 10, 2024. All data were fully de-identified prior to access and analysis, and the authors did not have access to personally identifiable information at any stage of the study. Descriptive statistics were used to summarize the characteristics of people with stroke and caregivers. Multivariable linear regression analysis was performed to identify factors associated with social participation. A total of 39 candidate predictors were initially considered based on the ICF framework and prior stroke cohort studies. Prior to model fitting, multicollinearity among candidate predictors was assessed using generalized variance inflation factors (GVIFs), and the adjusted GVIF values were within an acceptable range, indicating no evidence of problematic multicollinearity. To reduce model complexity and minimize the risk of overfitting, backward elimination was used to derive the final regression model for each functional subgroup. In addition, standard regression assumptions were evaluated using residual diagnostic plots. Missing data were handled using complete-case analysis. Among participants who met the inclusion criteria and completed the 12-month follow-up, those with missing values in variables required for the multivariable regression analyses were excluded from the final analytic sample. No imputation procedure was applied. A p-value of < 0.05 was considered statistically significant.

## Results

### Study population

Among the 10,636 stroke patients admitted, 8,010 consented to participate in the study. 2,881 people with stroke were excluded because they refused to follow up, did not contact the hospital, died and so on. Among the 5,129 stroke patients followed up at 12 months, 3,829 met the inclusion criteria. Of these 3,829 participants, 1,134 were excluded because of missing values in one or more variables required for the multivariable regression analyses. Therefore, the final analytic sample consisted of 2,695 participants and was based on complete-case analysis. A comparison of baseline characteristics between participants included in the final analysis and those excluded because of missing data showed statistically significant differences in age and baseline NIHSS, whereas sex and stroke type did not differ significantly (Table 2). However, the absolute differences in age and baseline NIHSS were small, suggesting that these statistically significant differences may have limited clinical significance.

**Table 2:** Baseline characteristics of included participants and participants excluded due to missing data.

Variable	Excluded due to missing data (n = 1,134)	Included in final analysis (n = 2,695)	p-value
Sex, n (%)			0.112
Male	673 (59.3)	1,675 (62.2)	
Female	461 (40.7)	1,020 (37.8)	
Age, years, mean ± SD	63.6 ± 12.5	62.1 ± 12.7	0.001
Stroke type, n (%)			0.477
Ischemic stroke	901 (79.5)	2,170 (80.5)	
Hemorrhagic stroke	233 (20.5)	525 (19.5)	
Baseline NIHSS, mean ± SD	3.5 ± 4.3	2.8 ± 4.0	<0.001

NIHSS, National Institutes of Health Stroke Scale; SD, standard deviation.

**Fig 2:**
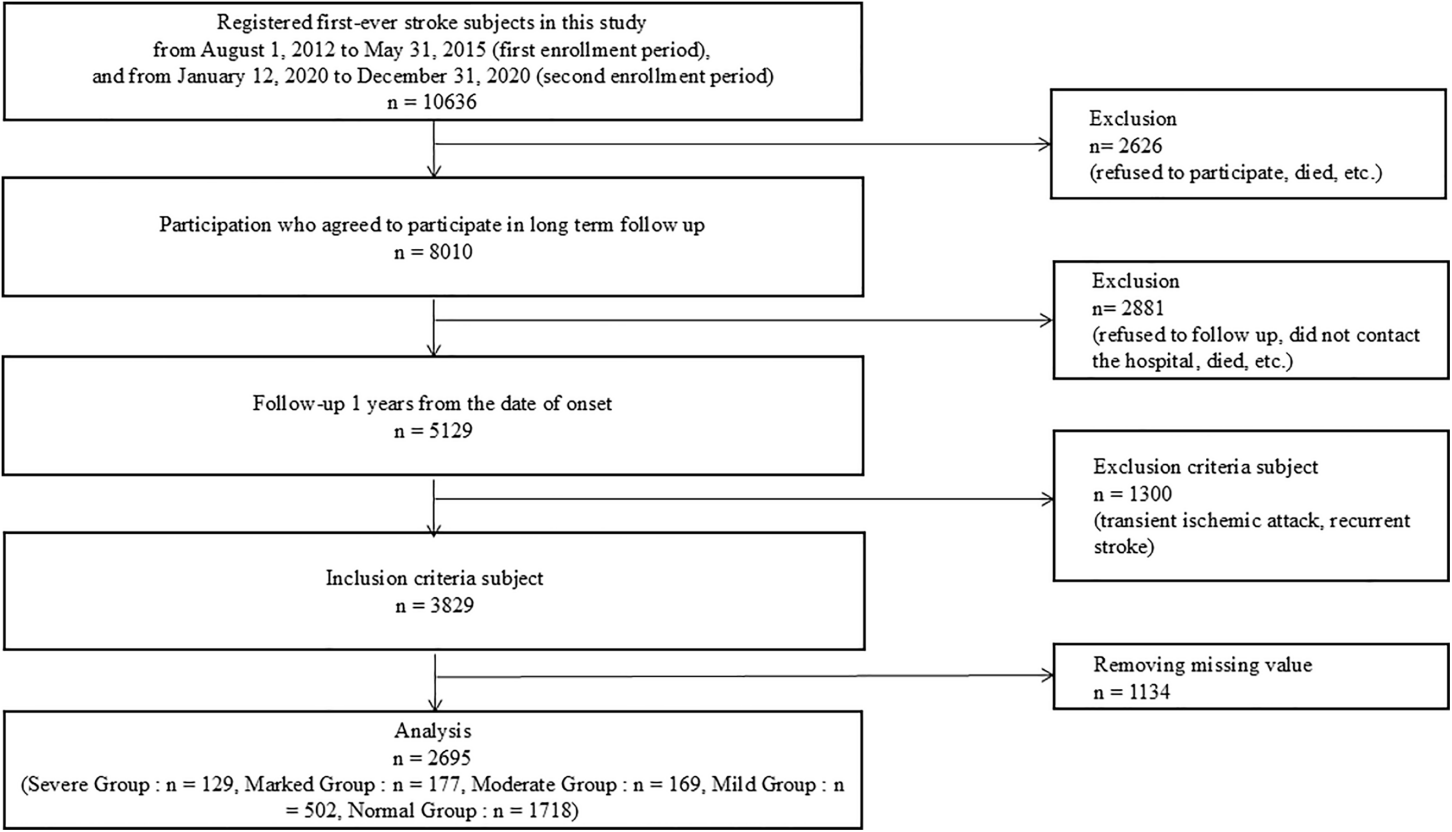
Flowchart of the study population.

Therefore, a total of 2,695 people with first ever stroke was included in the final analysis. Among the 2,695 patients, 129 were in the **Severe group (FMA < 50)**, 177 were in the **Marked group (50 ≤ FMA ≤ 84)**, 169 were in the **Moderate group (85 ≤ FMA ≤ 94)**, 502 were in the **Slight group (95 ≤ FMA ≤ 99)**, and 1,718 were in the **Unimpaired group (FMA = 100)** (Fig 2).

### Participant characteristics

The mean (SD) age of the Overall participants was 62.6 (12.7) years. Most were men (62.2%) and had a high school (32.6%) or higher (23.8%) education. Half of the participants had a specific religion (50.0%). Additional descriptive characteristics of people with stroke are shown in Table 3 and 4. The results of the interviews with 949 caregivers are shown in Table 5.

**Table 3:** Characteristics of persons with stroke according to personal factors, and body functions and structures.

Independent variables	Overall	FMA < 50 (Severe)	50 ≤ FMA ≤ 84 (Marked)	85 ≤ FMA ≤ 94 (Moderate)	95 ≤ FMA ≤ 99 (Slight)	FMA = 100 (Unimpaired)
	(N = 2695)	(N = 129)	(N = 177)	(N = 169)	(N = 502)	(N = 1718)
RNLI	84.4 ± 24.1	36.1 ± 27.2	53.7 ± 29.9	69.6 ± 24.8	85.3 ± 20.6	92.5 ± 14.9
Personal factors						
Age (years), mean (SD)	62.6 ± 12.7	62.6 ± 12.1	64.9 ± 11.0	66.6 ± 14.0	66.3 ± 11.5	60.9 ± 12.8
Sex, n (%)						
Male	1675 (62.2%)	80 (62.0%)	109 (61.6%)	86 (50.9%)	283 (56.4%)	1117 (65.0%)
Female	1020 (37.8%)	49 (38.0%)	68 (38.4%)	83 (49.1%)	219 (43.6%)	601 (35.0%)
Education, n (%)						
None	274 (10.2%)	14 (10.9%)	17 (9.6%)	22 (13.0%)	57 (11.4%)	164 (9.5%)
Primary education	447 (16.6%)	23 (17.8%)	40 (22.6%)	38 (22.5%)	149 (29.7%)	197 (11.5%)
Middle school education	455 (16.9%)	27 (20.9%)	36 (20.3%)	30 (17.8%)	80 (15.9%)	282 (16.4%)
High school education	878 (32.6%)	45 (34.9%)	45 (25.4%)	58 (34.3%)	137 (27.3%)	593 (34.5%)
University education	641 (23.8%)	20 (15.5%)	39 (22.0%)	21 (12.4%)	79 (15.7%)	482 (28.1%)
Religion, n (%)						
None	1348 (50.0%)	63 (48.8%)	81 (45.8%)	84 (49.7%)	267 (53.2%)	853 (49.7%)
Buddhism	582 (21.6%)	44 (34.1%)	33 (18.6%)	34 (20.1%)	91 (18.1%)	380 (22.1%)
Christianity	549 (20.4%)	13 (10.1%)	44 (24.9%)	39 (23.1%)	102 (20.3%)	351 (20.4%)
Catholic	194 (7.2%)	8 (6.2%)	17 (9.6%)	11 (6.5%)	36 (7.2%)	122 (7.1%)
Others	22 (0.8%)	1 (0.8%)	2 (1.1%)	1 (0.6%)	6 (1.2%)	12 (0.7%)
Smoking, yes, n (%)	263 (9.8%)	4 (3.1%)	8 (4.5%)	4 (2.4%)	52 (10.4%)	195 (11.4%)
Alcohol consumption, yes, n (%)	692 (25.7%)	5 (3.9%)	20 (11.3%)	20 (11.8%)	105 (20.9%)	542 (31.5%)
Stroke type, n (%)						
Ischaemic stroke	2170 (80.5%)	91 (70.5%)	132 (74.6%)	134 (79.3%)	421 (83.9%)	1392 (81.0%)
Haemorrhagic stroke	525 (19.5%)	38 (29.5%)	45 (25.4%)	35 (20.7%)	81 (16.1%)	326 (19.0%)
Body functions and structures						
Motor function (FMA; total affected side), mean (SD)	93.5 ± 17.4	26.1 ± 13.6	69.9 ± 10.7	90.7 ± 2.6	97.6 ± 1.0	100
Walking ability (FAC), n (%)						
Nonfunctional ambulator (0)	42 (1.6%)	30 (23.3%)	11 (6.2%)	1 (0.6%)	0 (0.0%)	0 (0%)
Ambulator, requires continuous manual contact (1)	48 (1.8%)	20 (15.5%)	13 (7.3%)	10 (5.9%)	1 (0.2%)	4 (0.2%)
Ambulator, requires intermittent touch to assist (2)	49 (1.8%)	17 (13.2%)	16 (9.0%)	6 (3.6%)	5 (1.0%)	5 (0.3%)
Dependent on supervision (3)	119 (4.4%)	29 (22.5%)	38 (21.5%)	17 (10.1%)	20 (4.0%)	15 (0.9%)
Independent, level surfaces only (4)	326 (12.1%)	22 (17.1%)	51 (28.8%)	52 (30.8%)	80 (15.9%)	121 (7.0%)
Independent, all surfaces (5)	2111 (78.3%)	11 (8.5%)	48 (27.1%)	83 (49.1%)	396 (78.9%)	1573 (91.6%)
Swallowing function (ASHA-NOMS), n (%)						
1	2 (0.1%)	0 (0%)	1 (0.6%)	0 (0%)	0 (0%)	1 (0.1%)
2	3 (0.1%)	2 (1.6%)	1 (0.6%)	0 (0%)	0 (0%)	0 (0%)
4	5 (0.2%)	2 (1.6%)	1 (0.6%)	1 (0.6%)	1 (0.2%)	0 (0%)
5	31 (1.2%)	11 (8.5%)	6 (3.4%)	6 (3.6%)	4 (0.8%)	4 (0.2%)
6	310 (11.5%)	31 (24.0%)	47 (26.6%)	28 (16.6%)	84 (16.7%)	120 (7.0%)
7	2344 (87.0%)	83 (64.3%)	121 (68.4%)	134 (79.3%)	413 (82.3%)	1593 (92.7%)
Cognitive function (K-MMSE)	26.8 ± 3.9	24.5 ± 5.2	24.7 ± 4.8	24.9 ± 5.2	26.2 ± 4.0	27.6 ± 3.3
Language function (K-FAST)	25.0 ± 6.4	20.8 ± 8.1	21.7 ± 7.4	22.4 ± 7.6	24.1 ± 6.6	26.2 ± 5.6
Depression (GDS-SF)	5.1 ± 4.0	8.6 ± 4.2	8.4 ± 4.1	7.5 ± 3.8	5.8 ± 4.0	4.1 ± 3.5
Psychological stress (PWI-SF)	15.7 ± 10.0	24.5 ± 11.0	24.1 ± 10.4	20.5 ± 8.6	18.5 ± 8.7	12.9 ± 9.1

ASHA-NOMS, American Speech Language Hearing Association National Outcomes Measurement System; FMA, Fugl-Meyer Assessment; FAC, Functional Ambulation Categories; GDS-SF, Geriatric Depression Scale-Short Form; K-FAST, Korean version of the Frenchay Aphasia Screening Test; K-MMSE, Korean version of the Mini Mental State Examination; PWI-SF, Psychosocial Wellbeing Index-Short Form; SD, standard deviation.

**Table 4:** Characteristics of persons with stroke according to activities and participation, and environmental factors.

Independent variables	Overall	FMA < 50 (Severe)	50 ≤ FMA ≤ 84 (Marked)	85 ≤ FMA ≤ 94 (Moderate)	95 ≤ FMA ≤ 99 (Slight)	FMA = 100 (Unimpaired)
	(N = 2695)	(N = 129)	(N = 177)	(N = 169)	(N = 502)	(N = 1718)
Activities and participation						
Disability or dependence (mRS), n (%)						
No symptoms at all (0)	964 (35.8%)	1 (0.8%)	2 (1.1%)	5 (3.0%)	108 (21.5%)	848 (49.4%)
No significant disability (1)	1121 (41.6%)	6 (4.7%)	22 (12.4%)	73 (43.2%)	289 (57.6%)	731 (42.5%)
Slight disability (2)	288 (10.7%)	10 (7.8%)	46 (26.0%)	51 (30.2%)	77 (15.3%)	104 (6.1%)
Moderate disability (3)	196 (7.3%)	47 (36.4%)	70 (39.5%)	28 (16.6%)	25 (5.0%)	26 (1.5%)
Moderate to severe disability (4)	88 (3.3%)	38 (29.5%)	30 (16.9%)	10 (5.9%)	3 (0.6%)	7 (0.4%)
Severe disability (5)	38 (1.4%)	27 (20.9%)	7 (4.0%)	2 (1.2%)	0 (0.0%)	2 (0.1%)
Activities of daily living (K-MBI), mean (SD)	94.9 ± 13.8	60.2 ± 24.6	79.0 ± 21.9	90.1 ± 15.7	97.6 ± 6.4	98.8 ± 5.6
Aerobic exercise, n (%)						
Never	860 (31.9%)	62 (48.1%)	81 (45.8%)	70 (41.4%)	158 (31.5%)	489 (28.5%)
Seldom (1–2 days per week)	101 (3.7%)	1 (0.8%)	3 (1.7%)	5 (3.0%)	18 (3.6%)	74 (4.3%)
Sometimes (3–5 days per week)	366 (13.6%)	5 (3.9%)	20 (11.3%)	23 (13.6%)	58 (11.6%)	260 (15.1%)
Very often (6–7 days per week)	1368 (50.8%)	61 (47.3%)	73 (41.2%)	71 (42.0%)	268 (53.4%)	895 (52.1%)
Driving, n (%)						
Not at all	1195 (44.3%)	60 (46.5%)	96 (54.2%)	101 (59.8%)	270 (53.8%)	668 (38.9%)
Not done before but done later	7 (0.3%)	0 (0%)	0 (0%)	1 (0.6%)	0 (0%)	6 (0.3%)
Done before but not later	485 (18.0%)	66 (51.2%)	61(34.5%)	44 (26.0%)	82(16.3%)	232(13.5%)
Consistently done	1008 (37.4%)	3 (2.3%)	20 (11.3%)	23 (13.6%)	150 (29.9%)	812 (47.3%)
Quality of life (EQ-5D), mean (SD)	0.8 ± 0.2	0.5 ± 0.3	0.6 ± 0.2	0.7 ± 0.2	0.9 ± 0.1	0.9 ± 0.1
Environmental factors						
Living arrangement, n (%)						
Living alone	321 (11.9%)	12 (9.3%)	23 (13.0%)	23 (13.6%)	72 (14.3%)	191 (11.1%)
Nuclear family	2223 (82.5%)	110 (85.3%)	138 (78.0%)	135 (79.9%)	394 (78.5%)	1446 (84.2%)
Extended family	151 (5.6%)	7 (5.4%)	16 (9.0%)	11 (6.5%)	36 (7.2%)	81 (4.7%)
Family support (FSI), mean (SD)	46.8 ± 8.1	46.4 ± 9.0	45.5 ± 8.9	46.0 ± 8.4	45.1 ± 7.4	47.6 ± 7.9
Employment status, yes, n (%)	971 (36.0%)	5 (3.9%)	17 (9.6%)	23 (13.6%)	145 (28.9%)	781 (45.5%)
Housing type						
Apartment	1215 (45.1%)	47 (36.4%)	78 (44.1%)	67 (39.6%)	207 (41.2%)	816 (47.5%)
Detached housing	1088 (40.4%)	58 (45.0%)	74 (41.8%)	73 (43.2%)	254 (50.6%)	629 (36.6%)
Multiunit housing	322 (11.9%)	19 (14.7%)	20 (11.3%)	21 (12.4%)	31 (6.2%)	231 (13.4%)
Others	70 (2.6%)	5 (3.9%)	5 (2.8%)	8 (4.7%)	10 (2.0%)	43 (2.4%)
Elevator use, yes, n (%)	1131 (42.0%)	48 (37.2%)	76 (42.9%)	65 (38.5%)	191 (38.0%)	751 (43.7%)
Inconvenient structure (entering or leaving home), yes, n (%)	479 (17.8%)	76 (58.9%)	77 (43.5%)	59 (34.9%)	107 (21.3%)	160 (9.3%)

EQ-5D, EuroQol-5 dimensions; FSI, Family Support Inventory; K-MBI, Korean version of the Modified Barthel Index; mRS, modified Rankin scale; SD, standard deviation.

**Table 5:** Characteristics of caregivers according to environmental factors.

Independent variables	Overall	FMA < 50 (Severe)	50 ≤ FMA ≤ 84 (Marked)	85 ≤ FMA ≤ 94 (Moderate)	95 ≤ FMA ≤ 99 (Slight)	FMA = 100 (Unimpaired)
	(N = 949)	(N = 86)	(N = 86)	(N = 100)	(N = 201)	(N = 476)
RNLI	74.3 ± 28.2	32.6 ± 23.0	46.1 ± 28.6	66.9 ± 26.4	77.4 ± 23.4	87.2 ± 18.3
Age (years), mean (SD)	56.6 ± 14.0	53.4 ± 14.8	56.2 ± 14.1	58.0 ± 13.8	56.0 ± 15.4	57.1 ± 13.2
Sex, n (%)						
Male	303 (31.9%)	28 (32.6%)	23 (26.7%)	34 (34.0%)	73 (36.3%)	145 (30.5%)
Female	646 (68.1%)	58 (67.4%)	63 (73.3%)	66 (66.0%)	128 (63.7%)	331 (69.5%)
Type of caregiver, n (%)						
Partner	583 (61.4%)	50 (58.1%)	50 (58.1%)	56 (56.0%)	110 (54.7%)	317 (66.6%)
Sons and daughters	270 (28.5%)	26 (30.2%)	27 (31.4%)	30 (30.0%)	69 (34.3%)	118 (24.8%)
Daughter in law	46 (4.8%)	0 (0%)	2 (2.3%)	8 (8.0%)	14 (7.0%)	22 (4.6%)
Parents	18 (1.9%)	6 (7.0%)	1 (1.2%)	3 (3.0%)	3 (1.5%)	5 (1.1%)
Certified Caregiver	3 (0.3%)	1 (1.2%)	0 (0%)	1 (1.0%)	0 (0.0%)	1 (0.2%)
Grandchild	5 (0.5%)	1 (1.2%)	1 (1.2%)	1 (1.0%)	2 (1.0%)	0 (0%)
Others	24 (2.5%)	2 (2.3%)	5 (5.8%)	1 (1.0%)	3 (1.5%)	13 (2.7%)
Vigorous intensity physical activity, n (%)						
Never	739 (77.9%)	73 (84.9%)	68 (79.1%)	84 (84.0%)	158 (78.6%)	356 (74.8%)
Seldom (1–2 days per week)	108 (11.4%)	8 (9.3%)	12 (14.0%)	8 (8.0%)	29 (14.4%)	51 (10.7%)
Sometimes (3–5 days per week)	67 (7.1%)	3 (3.5%)	4 (4.7%)	5 (5.0%)	10 (5.0%)	45 (9.5%)
Very often (6–7 days per week)	35 (3.7%)	2 (2.3%)	2 (2.3%)	3 (3.0%)	4 (2.0%)	24 (5.0%)
Moderate intensity physical activities, n (%)						
Never	716 (75.4%)	68 (79.1%)	63 (73.3%)	80 (80.0%)	157 (78.1%)	348 (10.1%)
Seldom (1–2 days per week)	96 (10.1%)	8 (9.3%)	9 (10.5%)	6 (6.0%)	17 (8.5%)	56 (11.8%)
Sometimes (3–5 days per week)	88 (9.3%)	7 (8.1%)	10 (11.6%)	9 (9.0%)	14 (7.0%)	48 (10.1%)
Very often (6–7 days per week)	49 (5.2%)	3 (3.5%)	4 (4.7%)	5 (5.0%)	13 (6.5%)	24 (5.0%)
Aerobic exercise, n (%)						
Never	304 (32.0%)	38 (44.2%)	22 (25.6%)	38 (38.0%)	68 (33.8%)	138 (29.0%)
Seldom (1–2 days per week)	90 (9.5%)	6 (7.0%)	9 (10.5%)	9 (9.0%)	24 (11.9%)	42 (8.8%)
Sometimes (3–5 days per week)	193 (20.3%)	14 (16.3%)	22 (25.6%)	20 (20.0%)	31 (15.4%)	106 (22.3%)
Very often (6–7 days per week)	362 (38.1%)	28 (32.6%)	33 (38.4%)	33 (33.0%)	78 (38.8%)	190 (39.9%)
Quality of life (EQ-5D), mean (SD)	0.9 ± 0.1	0.9 ± 0.1	0.9 ± 0.1	0.9 ± 0.1	0.9 ± 0.1	0.9 ± 0.1
Caregiver burden (CBI), mean (SD)	31.0 ± 14.2	43.4 ± 14.4	42.1 ± 13.8	35.1 ± 13.7	28.6 ± 13.4	26.9 ± 11.9

CBI, Caregiver Burden Inventory; EQ-5D, EuroQol-5 dimensions; PWI-SF, Psychosocial Wellbeing Index Short Form; SD, standard deviation.

### Social participation

At 12 months, the mean Reintegration to Normal Living Index (RNLI) score for all stroke patients was 84.4 (SD 24.1), with a 95% confidence interval of 83.5 to 85.3. The Severe group (FMA < 50) was 36.1 (27.2), the Marked group (50 ≤ FMA ≤ 84) was 53.7 (29.9), the Moderate group (85 ≤ FMA ≤ 94) was 69.6 (24.8), the Slight group (95 ≤ FMA ≤ 99) was 85.3 (20.6) and the Unimpaired group (FMA = 100) was 92.5 (14.9). This indicates that the more severe the patients’ disability, the lower their social participation.

### Factors affecting social participation at 12 months after stroke

The results of the multivariate regression analysis are shown in Table 6 and Table 7. Valid independent variables showed different results depending on the FMA score. These valid independent variables that positively or negatively affect social participation were classified according to the ICF framework and summarized in Table 8.

**Table 6:** Multivariate regression analysis of independent variables for social participation in persons with stroke.

Overall participants (n = 2695)	E	SE	*t*	*95% CI*	*Effect size*	*P*
Intercept	14.72	4.62	3.19	(5.66, 23.78)	–	<0.001
MMSE	0.46	0.10	4.65	(0.266, 0.653)	0.075	<0.001
K-FAST	0.18	0.06	2.83	(0.059, 0.298)	0.048	<0.001
K-MBI	0.48	0.04	11.11	(0.399, 0.570)	0.277	<0.001
EQ5D	31.69	2.57	12.34	(26.7, 36.7)	0.212	<0.001
Aerobic exercise+++	2.53	0.52	4.86	(1.51, 3.55)	0.105	<0.001
Absence of inconvenient home structure	1.48	0.66	2.25	(0.193, 2.77)	0.062	0.02
Not consuming alcohol	−1.35	0.54	−2.48	(−2.41, −0.289)	−0.056	0.01
GDS	−0.73	0.08	−9.12	(−0.881, −0.570)	−0.120	<0.001
PWI-SF	−0.20	0.03	−6.27	(−0.258, −0.135)	−0.082	<0.001
Unemployment	−1.34	0.53	−2.54	(−2.37, −0.304)	−0.056	0.01
*F* = 399.849 *p* < 0.001 Adjust R^2^ = 0.773						
FMA < 50 (Severe group, SVG) (n = 129)	E	SE	*t*	*95% CI*	*Effect size*	*P*
Intercept	−20.28	22.32	−0.91	(−64.47, 23.91)	–	0.37
MMSE	1.37	0.32	4.24	(0.729, 2.01)	0.261	<0.001
K-MBI	0.26	0.12	2.19	(0.025, 0.489)	0.232	0.03
EQ5D	15.68	7.46	2.10	(0.904, 30.5)	0.160	0.04
Aerobic exercise+	−61.95	16.71	−3.71	(−95.1, −28.8)	−2.28	<0.001
Absence of inconvenient home structure	7.75	3.10	2.50	(1.61, 13.9)	0.285	0.01
Type of housing + :	7.15	3.42	2.09	(0.367, 13.9)	0.263	0.04
GDS	−1.38	0.36	−3.87	(−2.09, −0.674)	−0.215	<0.001
Driving+	−19.49	9.03	−2.16	(−37.4, −1.60)	−0.716	0.03
*F* = 18.004 *p* < 0.001 Adjust R^2^ = 0.716						
50 ≤ FMA ≤ 84 (Marked group, MKG) (n = 177)	E	SE	*t*	*95% CI*	*Effect size*	*P*
Intercept	24.41	11.95	2.04	(0.82, 48.00)	–	0.04
K-MBI	0.34	0.10	3.53	(0.149, 0.527)	0.248	<0.001
EQ5D	32.78	9.78	3.35	(13.5, 52.1)	0.240	<0.001
Aerobic exercise+++	8.36	3.32	2.52	(1.80, 14.9)	0.280	0.01
GDS	−1.48	0.45	−3.26	(−2.37, −0.583)	−0.200	<0.001
Driving++	−11.78	5.30	−2.22	(−22.3, −1.32)	−0.394	<0.001
*F* = 25.573 *p* < 0.001 Adjust R^2^ = 0.645						
85 ≤ FMA ≤ 94 (Moderate group, MDG) (n = 169)	E	SE	*t*	*95% CI*	*Effect size*	*P*
Intercept	−19.44	19.17	−1.01	(−57.30, 18.42)	–	0.31
K-FAST	0.47	0.18	2.65	(0.120, 0.181)	0.144	0.01
K-MBI	0.86	0.16	5.41	(0.543, 1.17)	0.541	<0.001
EQ5D	31.39	8.82	3.56	(14.0, 48.8)	0.226	<0.001
GDS	−1.06	0.35	−2.98	(−1.76, −0.355)	−0.163	<0.001
PWI-SF	−0.38	0.16	−2.36	(−0.701, −0.062)	−0.133	0.02
Unemployment	−8.13	3.28	−2.47	(−14.6, −1.64)	−0.328	<0.001
*F* = 30.070 *p* < 0.001 Adjust R^2^ = 0.692						
95 ≤ FMA ≤ 99 (Slight group, SLG) (n = 502)	E	SE	*t*	*95% CI*	*Effect size*	*P*
Intercept	−7.48	17.95	−0.42	(−42.75, 27.79)	–	0.68
MMSE	0.73	0.18	4.12	(0.384, 1.08)	0.141	<0.001
K-MBI	0.89	0.15	5.75	(0.586, 1.19)	0.277	<0.001
EQ5D	31.27	6.92	4.52	(17.7, 44.9)	0.176	<0.001
Aerobic exercise++	3.68	1.86	1.98	(0.031, 7.33)	0.179	0.05
Aerobic exercise+++	4.18	1.24	3.38	(1.75, 6.62)	0.203	<0.001
Absence of inconvenient home structure	3.48	1.47	2.37	(0.589, 6.36)	0.169	0.02
Absence of elevator	2.58	1.19	2.17	(0.241, 4.92)	0.125	0.03
Lower education levels	−3.81	1.54	−2.48	(−6.83, −0.786)	−0.185	0.01
GDS	−0.49	0.20	−2.49	(−0.879, −0.103)	−0.096	0.01
PWI-SF	−0.33	0.09	−3.74	(−0.501, −0.156)	−0.138	<0.001
Unemployment	−3.11	1.34	−2.33	(−5.73, −0.483)	−0.151	0.02
*F* = 42.592 *p* < 0.001 Adjust R^2^ = 0.666						
FMA = 100 (Unimpaired group, NMG) (n = 1718)	E	SE	*t*	*95% CI*	*Effect size*	*P*
Intercept	−35.57	11.67	−3.05	(−58.46, −12.68)	–	<0.001
MMSE	0.64	0.11	5.88	(0.430, 0.859)	0.142	<0.001
Swallowing function+	19.64	9.14	2.15	(1.72, 37.6)	1.32	0.03
Swallowing function++	18.58	9.10	2.04	(0.738, 36.4)	1.25	0.04
K-MBI	0.71	0.07	10.10	(0.570, 0.844)	0.264	<0.001
EQ5D	40.80	3.52	11.60	(33.9, 47.7)	0.244	<0.001
Aerobic exercise+++	1.12	0.52	2.17	(0.108, 2.13)	0.075	0.03
GDS	−0.58	0.08	−7.12	(−0.742, −0.421)	−0.135	<0.001
PWI-SF	−0.10	0.03	−3.22	(−0.169, −0.041)	−0.064	<0.001
*F* = 122.966 *p* < 0.001 Adjust R^2^ = 0.630						

Adjust R2, adjusted R-squared; ASHA-NOMS, American Speech Language Hearing Association National Outcomes Measurement System; E, estimate; EQ-5D, EuroQol-5 dimensions; F, F-value; GDS, Geriatric Depression Scale; K-FAST, Korean version of the Frenchay Aphasia Screening Test; K-MBI, Korean version of the Modified Barthel Index; MMSE, Mini-Mental State Examination; p, p-value; PWI-SF, Psychosocial Wellbeing Index-Short Form; SE, standard error; t, t-value.

Aerobic exercise + : 1–2 days

Aerobic exercise++: 3–5 days

Aerobic exercise+++: 6–7 days

Driving + : Done before but not later

Driving++: not at all before or after stroke.

Type of housing + : Detached housing

Type of housing++: multi-unit housing

Swallowing function + : ASHA-NOMS 6

**Table 7:** Multivariate regression analysis of independent variables of caregivers affecting patients’ social participation.

Overall participants (n = 2695)	E	SE	*t*	*95% CI*	*Effect size*	*P*
Intercept	113.38	10.81	10.49	(92.18, 134.58)	–	<0.001
Female caregiver	4.04	1.71	2.36	(0.778, 7.50)	0.147	0.02
Caregiver’s Age	−0.16	0.07	−2.29	(−0.300, −0.022)	−0.080	0.02
CBI	−1.06	0.06	−18.90	(−1.18, −0.955)	−0.535	<0.001
Caregiver Type – Certified caregiver	−34.07	13.24	−2.57	(−59.6, −7.65)	−1.19	0.01
Caregiver Type – Grandchild	−26.79	10.49	−2.55	(−46.8, −5.56)	−0.927	0.01
Caregiver Type – Sons and Daughters	−16.46	2.15	−7.65	(−20.7, −12.3)	−0.459	<0.001
Caregiver Type – Daughter-in-law	−12.85	3.71	−3.46	(−20.3, −5.68)	−0.459	<0.001
Caregiver not engaging in exercise	−6.79	2.79	−2.43	(−12.2, −1.12)	−0.236	0.02
*F* = 33.198 *p* < 0.001 Adjust R^2^ = 0.352						
FMA < 50 (Severe group, SVG) (n = 129)	E	SE	*t*	*95% CI*	*Effect size*	*P*
Intercept	106.99	15.44	6.93	(76.43, 137.55)	–	<0.001
Caregiver’s Age	−0.64	0.19	−3.28	(−1.03, −0.251)	−0.409	<0.001
CBI	−0.68	0.16	−4.29	(−0.99, −0.362)	−0.423	<0.001
Caregiver Type – Sons and Daughters	−19.14	6.21	−3.08	(−31.5, −6.76)	−0.831	<0.001
*F* = 4.302 *p* < 0.001 Adjust R^2^ = 0.299						
50 ≤ FMA ≤ 84 (Marked group, MKG) (n = 177)	E	SE	*t*	*95% CI*	*Effect size*	*P*
Intercept	73.92	12.12	6.10	(50.00, 97.84)	–	<0.001
Aerobic exercise ++	25.91	11.96	2.17	(2.11, 49.7)	0.905	0.03
CBI	−0.77	0.21	−3.73	(−1.18, −0.358)	−0.369	<0.001
*F* = 5.500 *p* < 0.001 Adjust R^2^ = 0.175						
85 ≤ FMA ≤ 94 (Moderate group, MDG) (n = 169)	E	SE	*t*	*95% CI*	*Effect size*	*P*
Intercept	121.12	17.72	6.83	(86.13, 156.11)	–	<0.001
CBI	−0.49	0.18	−2.71	(−0.855, −0.132)	−0.256	0.01
Caregiver Type – Sons and Daughters	−32.53	6.87	−4.73	(−46.2, −18.9)	−1.23	<0.001
*F* = 3.367 *p* < 0.001 Adjust R^2^ = 0.208						
95 ≤ FMA ≤ 99 (Slight group, SLG) (n = 502)	E	SE	*t*	*95% CI*	*Effect size*	*P*
Intercept	96.55	18.97	5.09	(59.28, 133.82)	–	<0.001
Caregiver’s Age	−0.29	0.13	−2.29	(−0.545, −0.040)	−0.192	0.02
CBI	−0.84	0.11	−7.34	(−1.06, −0.612)	−0.480	<0.001
Caregiver Type – Sons and Daughters	−13.40	4.07	−3.29	(−21.4, −5.37)	−0.573	<0.001
*F* = 11.967 *p* < 0.001 Adjust R^2^ = 0.305						
FMA = 100 (Unimpaired group, NMG) (n = 1718)	E	SE	*t*	*95% CI*	*Effect size*	*P*
Intercept	93.14	11.28	8.26	(71.02, 115.26)	–	<0.001
High caregiver quality of life	28.16	10.11	2.79	(8.30, 48.0)	0.120	0.01
Caregiver’s Age	−0.23	0.07	−3.20	(−0.364, −0.087)	−0.162	<0.001
CBI	−0.52	0.06	−8.14	(−0.648, −0.396)	−0.340	<0.001
Caregiver Type – Sons and Daughters	−14.73	2.08	−7.09	(−18.8, −10.6)	−0.804	<0.001
Caregiver Type – Daughter-in-law	−17.10	3.71	−4.61	(−24.1, −9.81)	−0.934	<0.001
*F* = 20.600 *p* < 0.001 Adjust R^2^ = 0.248						

Adjust R^2^, adjusted R-squared; CBI, Caregiver Burden Inventory; E, estimate; *F*, *F*-value; *p*, *p*-value; SE, standard error; *t*, *t*-value.

Aerobic exercise++: 3–5 days

**Table 8:** Factors classified according to ICF’s 4 frame that affect social participation based on FMA score.

		FMA score					
ICF’s 4 frame		Overall	FMA < 50	50 ≤ FMA ≤ 84	85 ≤ FMA ≤ 94	95 ≤ FMA ≤ 99	FMA = 100
**Personal related factors**	**Positive relations**	–	–	–	–	–	–
**Body functions and Structures factors**		- MMSE - K-FAST	- MMSE	–	- K-FAST	- MMSE	- MMSE - ASHA-NOMS
**Activities and Participation factors**		- K-MBI - EQ5D - Aerobic exercise(6–7days)	- K-MBI - EQ5D	- K-MBI - EQ5D - Aerobic exercise (6–7days)	- K-MBI - EQ5D	- K-MBI - EQ5D - Aerobic exercise (3–5days < 6–7days)	- K-MBI - EQ5D - Aerobic exercise (6–7days)
**Environmental factors**		- Absence of inconvenient home structures - Having a female caregiver	- Absence of inconvenient home structures, - Living in a detached house	- Caregiver aerobic exercise (3–5 days)	–	- Absence of inconvenient home structures - Absence of an elevator	- High caregiver quality of life
**FMA score** **ICF’s 4 frame**		**Overall**	**FMA < 50**	**50 ≤ FMA ≤ 84**	**85 ≤ FMA ≤ 94**	**95 ≤ FMA ≤ 99**	**FMA = 100**
**Personal related factors**	**Negative relations**	- Absence of alcohol consumption	–	–	–	- Lower education levels	–
**Body functions and Structures factors**		- GDS - PWI-SF	- GDS	- GDS	- GDS - PWI-SF	- GDS - PWI-SF	- GDS - PWI-SF
**Activities and Participation factors**		Unemployment -	- Cessation of driving post-stroke - Aerobic exercise(1–2days)	- Non-driving pre- and post-stroke	Unemployment -	- Unemployment	–
**Environmental factors**		– - Older caregiver age - CBI - Being cared for by certified caregiver, grandchild, sons and daughters, or daughter-in-law - Caregiver not engaging in exercise	- Older caregiver age - CBI - Being cared for by sons and daughters	- CBI	– - CBI - Being cared for by sons and daughters	– - Older caregiver age - CBI - Being cared for by sons and daughters	- Older caregiver age - CBI - Being cared for by a daughter-in-law or sons and daughters

#### Swallowing function++: ASHA-NOMS 7

- **Overall Participants group (n = 2,695)**

The analysis of the **Overall participants group** (n = 2,695) identified significant predictors of social participation. Among these, EQ5D(E = 31.69; SE = 2.57; p < 0.001), female caregiver(E = 4.04; SE = 1.71; p = 0.02), engaging in aerobic exercise 6–7 days a week(E = 2.53; SE = 0.52; p < 0.001), absence of inconvenient home structure(E = 1.48; SE = 0.66; p = 0.02), K-MBI(E = 0.48; SE = 0.04; p < 0.001), MMSE(E = 0.46; SE = 0.10; p < 0.001) and K-FAST(E = 0.18; SE = 0.06; p < 0.001) were found to be positive predictors of social participation, listed in order of impact.

Conversely, receiving care from certified caregiver(E = −34.07; SE = 13.24; p = 0.01), receiving care from grandchild(E = −26.79; SE = 10.49; p = 0.01), receiving care from sons and daughters(E = −16.46; SE = 2.15; p < 0.001), caregiver not engaging in exercise(E = −6.79; SE = 2.79; p = 0.02), abstinence from alcohol(E = −1.35; SE = 0.54; p = 0.01), unemployment(E = −1.34; SE = 0.53; p = 0.01), CBI(E = −1.06; SE = 0.06; p < 0.001), GDS(E = −0.73; SE = 0.08; p < 0.001), PWI-SF(E = −0.20; SE = 0.03; p < 0.001) and caregiver’s old age(E = −0.16; SE = 0.07; p = 0.02) were identified as negative predictors and this is ranked by their influence on social participation.

Notably, emotional factors such as depression (GDS) and psychosocial stress (PWI-SF) emerged as predominant negative predictors across nearly all functional levels. This indicates that a patient’s emotional state serves as a critical internal determinant of reintegration, exerting a significant influence regardless of the severity of physical impairment.

The explanatory power (R2) of the final analysis was 77.3% for the characteristics of all participations with stroke and 35.2% for the characteristics of caregivers.

- **Severe group (FMA<50, n = 129)**

The analysis of the Severe group (n = 129) identified significant predictors of social participation. Among these, MMSE(E = 1.37; SE = 0.32; p < 0.001), K-MBI(E = 0.26; SE = 0.12; p = 0.03), EQ5D(E = 15.68; SE = 7.46; p = 0.04), absence of inconvenient home structure(E = 7.75; SE = 3.10; p = 0.01), Living in a detached house (E = 7.15; SE = 3.42; p = 0.04) were found to be positive predictors of social participation, listed in order of impact.

Conversely, engaging in aerobic exercise 1−2 days a week(E = −61.95; SE = 16.71; p < 0.001), Cessation of driving post-stroke(E = −19.49; SE = 9.03; p = 0.03), Being cared for by sons and daughters(E = −19.14; SE = 6.21; p < 0.001), GDS(E = −1.38; SE = 0.36; p < 0.001), CBI(E = −0.68; SE = 0.16; p < 0.001), Older caregiver age(E = −0.64; SE = 0.19; p < 0.001) were identified as negative predictors and this is ranked by their influence on social participation.

The explanatory power (R2) of the final analysis was 71.6% for the characteristics of severe group participations with stroke and 29.9% for the characteristics of caregivers.

- **Marked group (50≤FMA≤84, n = 177)**

The analysis of the Marked group (n = 177) identified significant predictors of social participation. Among these, EQ5D(E = 32.78; SE = 9.78; p < 0.001), caregiver engaging in aerobic exercise 3–5 days a week(E = 25.91; SE = 11.96; p = 0.03), engaging in aerobic exercise 6–7 days a week(E = 8.36; SE = 3.32; p = 0.01) and K-MBI(E = 0.34; SE = 0.10; p < 0.001) were found to be positive predictors of social participation, listed in order of impact.

Conversely, GDS(E = −1.48; SE = 0.45; p < 0.001), CBI(E = −0.77; SE = 0.21; p < 0.001), Non-driving pre- and post-stroke(E = −11.78; SE = 5.30; p < 0.001) were identified as negative predictors and this is ranked by their influence on social participation.

The explanatory power (R2) of the final analysis was 64.5% for the characteristics of all participations with stroke and 17.5% for the characteristics of caregivers.

- **Moderate group (85≤FMA≤94, n = 169)**

The analysis of the Moderate group (n = 169) identified significant predictors of social participation. Among these, EQ5D(E = 31.39; SE = 8.82; p < 0.001), K-MBI(E = 0.86; SE = 0.16; p < 0.001) and K-FAST(E = 0.47; SE = 0.18; p = 0.01) were found to be positive predictors of social participation, listed in order of impact.

Conversely, receiving care from sons and daughters(E = −32.53; SE = 6.87; p < 0.001), unemployment(E = −8.13; SE = 3.28; p < 0.001), GDS(E = −1.06; SE = 0.35; p < 0.001), CBI(E = −0.49; SE = 0.18; p = 0.01) and PWI-SF(E = −0.38; SE = 0.16; p = 0.02) were identified as negative predictors and this is ranked by their influence on social participation.

The explanatory power (R2) of the final analysis was 69.2% for the characteristics of all participations with stroke and 20.8% for the characteristics of caregivers.

- **Slight group (95≤FMA≤99, n = 502)**

The analysis of the Slight group (n = 502) identified significant predictors of social participation. Among these, EQ5D(E = 31.27; SE = 6.92; p < 0.001), engaging in aerobic exercise 6–7 days a week(E = 4.18; SE = 1.24; p < 0.001), engaging in aerobic exercise 3–5 days a week(E = 3.68; SE = 1.86; p = 0.05), absence of inconvenient home structure(E = 3.48; SE = 1.47; p = 0.02), absence of an elevator(E = 2.58; SE = 1.19; p = 0.03) and K-MBI(E = 0.89; SE = 0.15; p < 0.001), MMSE(E = 0.73; SE = 0.18; p < 0.001) were found to be positive predictors of social participation, listed in order of impact.

Conversely, receiving care from sons and daughters(E = −13.40; SE = 4.07; p < 0.001), lower education levels(E = −3.81; SE = 1.54; p = 0.01), unemployment(E = −3.11; SE = 1.34; p = 0.02), CBI(E = −0.84; SE = 0.11; p < 0.001), GDS(E = −0.49; SE = 0.20; p = 0.01), PWI-SF(E = −0.33; SE = 0.09; p < 0.001) and caregiver’s old age(E = −0.29; SE = 0.13; p = 0.02) were identified as negative predictors and this is ranked by their influence on social participation.

The explanatory power (R2) of the final analysis was 66.6% for the characteristics of all participations with stroke and 30.5% for the characteristics of caregivers.

- **Unimpaired group (FMA=100, n = 1718)**

The analysis of the Unimpaired group (n = 1718) identified significant predictors of social participation. Among these, EQ5D(E = 40.80; SE = 3.52; p < 0.001), high caregiver quality of life(E = 28.16; SE = 10.11; p = 0.01), ASHA-NOMS level 6 swallowing function(E = 19.64; SE = 9.14; p = 0.03), ASHA-NOMS level 7 swallowing function(E = 18.58; SE = 9.10; p = 0.04), engaging in aerobic exercise 6–7 days a week(E = 1.12; SE = 0.52; p = 0.03), K-MBI(E = 0.71; SE = 0.07; p < 0.001) and MMSE(E = 0.64; SE = 0.11; p < 0.001) were found to be positive predictors of social participation, listed in order of impact.

Conversely, receiving care from daughter-in-law(E = −17.10; SE = 3.71; p < 0.001), receiving care from sons and daughters(E = −14.73; SE = 2.08; p < 0.001), GDS(E = −0.58; SE = 0.08; p < 0.001), CBI(E = −0.52; SE = 0.06; p < 0.001), caregiver’s old age(E = −0.23; SE = 0.07; p < 0.001) and PWI-SF(E = −0.10; SE = 0.03; p < 0.001) were identified as negative predictors and this is ranked by their influence on social participation.

**Fig 3:**
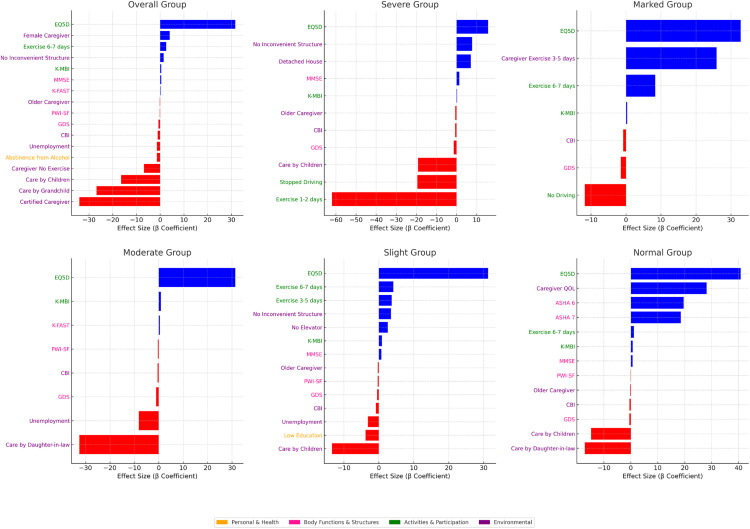
ICF-Based Predictors of Social Participation in Stroke Patients by Functional Level.

The explanatory power (R2) of the final analysis was 63.0% for the characteristics of all participations with stroke and 24.8% for the characteristics of caregivers (Fig 3).

Bar plots show significant predictors of social participation 12 months after stroke, stratified by Fugl-Meyer Assessment (FMA) functional levels. Predictors are ordered by the absolute value of their effect size. Blue bars indicate positive associations; red bars indicate negative associations. The color of each label on the y-axis corresponds to its classification according to the International Classification of Functioning, Disability and Health (ICF) framework

## Discussion

This study analyzed the factors influencing social participation after stroke according to functional level, based on the International Classification of Functioning, Disability, and Health (ICF) framework. Most of the significant predictors were associated with Activities and Participation and Environmental Factors, rather than Body Functions and Structures. Importantly, these domains are often modifiable in clinical practice. This highlights the practical value of targeting not only physical recovery, but also interventions that enhance independence, support community reintegration, and reduce environmental barriers. Rehabilitation strategies should therefore adopt a multidimensional approach, incorporating tailored interventions based on functional level. Clinicians should prioritize individualized goal setting that addresses activity limitations and environmental constraints, which are often more amenable to change than structural impairments.

K-MBI (functional independence) and EQ-5D (health-related quality of life) consistently had positive effects on social participation across all groups. These findings confirm that functional independence and overall well-being [30] serve as the foundation for successful social reintegration. On the other hand, depressive symptoms (GDS) and caregiver burden (CBI) were major negative predictors across all groups, suggesting the importance of early mental health screening [31,32] and caregiver support interventions [33]. This finding underscores the conceptual significance of emotional well-being as a prerequisite for social participation. Even when stroke survivors achieve functional independence, unresolved depression and high stress levels act as ‘invisible barriers’ that diminish their motivation and psychological readiness to engage in social activities. Therefore, our results suggest that emotional distress can be as restrictive as physical disability. Crucially, addressing this emotional barrier requires long-term vigilance rather than a one-time assessment because post-stroke depressive symptoms fluctuate significantly across the 1-year trajectory of recovery [34]. Consequently, rehabilitation strategies must move beyond a single early screening to implement a proactive, ongoing screening approach throughout the entire continuum of care. Based on these continuous assessments, comprehensive and timely psychosocial interventions should be implemented to facilitate effective long-term social integration.

In addition to these general trends, the factors influencing social participation differed by functional level. In the Severe group (FMA < 50), due to substantial physical limitations, environmental factors and caregiver characteristics had the greatest impact on participation. Improved accessibility of the residential environment [35,36] and living in a detached house were associated with better participation, while caregiving by sons and daughters, older caregivers, and high caregiver burden were identified as barriers [33]. These findings suggest that rehabilitation for severely impaired patients should not be limited to physical recovery alone, but should adopt a multidimensional approach that includes environmental modifications [6,7,37], caregiver support [33], and home adaptations to enhance overall living conditions [35,36]. Crucially, these environmental interventions should be led by occupational therapists (OTs), who can perform professional home safety assessments to tailor modifications to the survivor’s specific physical limitations and domestic layout. Rehabilitation physicians should actively consider both the assessment of the home environment and the provision of resources to alleviate caregiver stress. Although behavioral factors such as engaging in aerobic exercise once or twice a week or driving cessation were also associated with reduced participation, these may be less accessible or modifiable given the severe functional limitations in this group.

In the Marked group (FMA 50–84), aerobic exercise was identified as a significant positive factor influencing social participation. This group often experiences reduced physical efficiency due to neurological damage and muscle weakness, along with diminished cardiopulmonary function and chronic fatigue. As a result, fatigue easily accumulates during physical activities, which can limit social participation. Therefore, aerobic exercise plays a crucial rehabilitative role by improving cardiopulmonary endurance and muscular strength, helping patients overcome these physical limitations and engage more actively in social activities [38]. Additionally, caregiver participation in aerobic exercise reduces caregiver burden and improves their physical and emotional health, thereby indirectly enhancing the quality of patient care and promoting patient social participation. Meanwhile, patients who had never driven prior to their stroke and remain unable to drive afterward tend to have lower levels of social participation. This likely reflects pre-existing mobility limitations, lower socioeconomic status, and limited access to transportation, among other environmental disadvantages. Clinically, driving history can serve as a useful early indicator to identify patients at risk of social isolation [39]. Based on this, targeted interventions such as community transportation services, assistive mobility devices, and tailored social participation programs are necessary. Addressing these pre-existing mobility limitations is essential to prevent long-term declines in social participation within this subgroup.

In the Moderate group (FMA 85–94), patients generally demonstrate substantial motor recovery and are functionally independent in performing most daily and social activities. However, residual language impairments continued to be a significant barrier to social participation in this group [40]. These non-motor deficits are often overlooked, particularly in patients who appear physically recovered, yet such limitations can critically hinder interpersonal relationships and community reintegration. In this context, speech and language therapy remains clinically important—not merely for improving expressive and receptive skills, but also for enhancing social confidence and facilitating meaningful participation in everyday life. Furthermore, unemployment was identified as a negative predictor of social participation [41]. Although patients in this group are physically capable of returning to work, delayed vocational reintegration may occur due to insufficient support for cognitive and communicative functions. Thus, individualized vocational rehabilitation programs, tailored to the patient’s residual impairments, are essential to promote re-employment and support broader community integration. Occupational therapy plays a pivotal role here, as OTs provide job analysis and workplace adaptations that facilitate the transition from clinical recovery to actual vocational reintegration. Additionally, caregiving by sons, daughters, or daughters-in-law was associated with decreased participation. This finding warrants a deeper sociocultural interpretation of the East Asian familial structure, as requested by the clinical context of this study. While traditionally expected to be primary caregivers in Korea, daughters-in-law may experience higher psychological distance and interpersonal tension compared to blood relatives. This compulsory altruism(performing caregiving duties out of social obligation rather than personal bond) can lead to hidden caregiver burnout, which in turn diminishes the quality of emotional support and social encouragement provided to the survivor. Furthermore, adult children often exhibit ‘overprotective’ behaviors, restricting the patient’s autonomy and community outings to prevent potential injuries. Such dynamics reduce opportunities for the patient to act independently while simultaneously increasing caregiver stress due to role conflict between professional and caregiving responsibilities. Therefore, family-centered psychosocial interventions including caregiver education, counseling, and stress management programs are crucial. These strategies aim to alleviate caregiver burden and create an environment that encourages patients to participate more autonomously in both daily and community activities.

In the Slight group (FMA 95–99), regular aerobic exercise (6–7 days per week) was identified as the most influential factor in promoting social participation. While these patients exhibit near-normal motor abilities, subtle limitations may remain in endurance, balance confidence, or psychological readiness for community reintegration. In such high-functioning individuals, aerobic exercise not only maintains physical health but also plays a therapeutic role by enhancing mood regulation, self-efficacy, and social motivation, thereby fostering real-world participation. Unlike in lower-functioning groups, where physical limitations are the primary barrier to exercise, in this group, lack of participation may stem more from psychosocial or environmental factors. These findings suggest that aerobic exercise should be emphasized not only as a fitness intervention but as a structured component of social rehabilitation. Clinicians should provide tailored exercise prescriptions with motivational strategies, and where appropriate, engage caregivers to support adherence. Programs promoting consistent participation—ideally ≥3 days per week—can function as both physical and social catalysts for reintegration. Environmental factors also played a role in participation. Interestingly, the absence of elevators or structural accessibility was positively associated with higher social participation [35,36]. This may be because patients in this group possess sufficient physical ability to benefit from mild daily challenges, such as stair climbing or walking longer distances, which naturally support more active and autonomous living. Clinicians should avoid over-accommodating environments in high-functioning patients and instead encourage safe, mobility-promoting settings that maintain activity.

In contrast, low educational attainment [9] and unemployment [41] were associated with decreased participation. Even among patients with near-complete motor recovery, limited cognitive resources, economic security, and access to social capital may hinder opportunities for community engagement. Therefore, it is crucial to identify such barriers early and integrate community-based education, peer support systems, and vocational services into long-term rehabilitation planning for this group.

Patients in the Slight group retain the functional capacity for full community reintegration, but their participation can be limited by psychosocial and contextual factors. Multidimensional interventions—including aerobic activity, environmental assessment, educational support, and vocational engagement—are essential to fully leverage their potential for sustained social recovery.

In the Unimpaired group (FMA = 100), swallowing function was significant positive predictor of social participation [42]. Although these patients show complete motor recovery, subtle swallowing impairments may persist and hinder participation in daily social contexts—especially shared meals, which are central to interpersonal engagement. Dysphagia in high-functioning individuals often goes unnoticed, yet it can cause discomfort, fear of aspiration, and social embarrassment, leading to avoidance of communal eating and increased isolation. Clinically, this highlights the importance of routinely assessing and addressing swallowing function, even in patients with full physical independence. Interventions such as swallowing therapy, dietary adjustments, and compensatory techniques should be incorporated into care plans to reduce hidden barriers and support full community reintegration.

Overall, these findings emphasize the need for individualized, function-based rehabilitation strategies that go beyond physical recovery to address psychosocial, communicative, and environmental barriers to social participation across all levels of stroke severity.

### Clinical implications

Function-based individualized interventions are essential for effective stroke rehabilitation. In addition to physical recovery, attention to mental health, environment, and caregiver systems enhances reintegration and overall quality of life [35].

### Public Health and policy implications

Beyond clinical practice, these findings offer broader implications for health systems. It is crucial to distinguish rehabilitation goals by timing: while early-stage interventions prioritize neuroplasticity and structural recovery, the focus at the 12-month mark—a period where neurological recovery typically reaches a plateau and social participation patterns tend to stabilize—must shift toward maximizing social integration. Currently, stroke rehabilitation policies in Korea are predominantly hospital-centered; however, our results underscore the necessity of a transition toward an integrated Community-Based Rehabilitation (CBR) system.

This transition must be tailored to the survivor’s functional severity. For the Severe group, who continue to face significant physical limitations, public health initiatives should prioritize environmental modifications and caregiver support systems. In this context, Occupational Therapists (OTs) play a pivotal role by conducting professional home-safety assessments to remove physical barriers and enhance patient independence, thereby directly alleviating the caregiving burden. This aligns with the findings of the Restore4Stroke cohort [9], which indicated that long-term societal costs are largely driven by informal care. Therefore, it is essential to expand respite care services to prevent caregiver burnout from becoming a secondary barrier to the survivor’s participation.

Conversely, for higher-functioning survivors, policy efforts should shift toward addressing ‘invisible barriers.’ The expertise of OTs should be utilized for cognitive-vocational rehabilitation and task adaptation, ensuring these individuals can successfully return to work and their social roles. Ultimately, health systems must secure dedicated funding to expand the community-based OT workforce, bridging the gap between hospital-centered medical care and participation-focused social welfare.

### Limitations

This study has several limitations. First, its cross-sectional design precludes causal inferences. Second, social participation was assessed using self-report measures, which may be subject to response bias. Third, variation in group sizes based on functional level may have affected statistical power, particularly in smaller groups. Lastly, some psychosocial variables could not be analyzed due to missing data. Furthermore, participants with incomplete data were excluded from the final analysis, which may have introduced some selection bias. In our comparison of available baseline characteristics, included and excluded participants differed significantly in age and baseline NIHSS, whereas sex and stroke type did not. However, the absolute differences in age and baseline NIHSS were small, suggesting that any resulting bias is unlikely to have materially affected the overall interpretation of the findings. Additionally, since this research was conducted within a specific regional cohort in South Korea, the findings—particularly regarding caregiver dynamics and sociocultural factors—may have limited generalizability to other global populations with different family structures and healthcare systems. Finally, while the RNLI is a well-validated tool, its reliance on self-reporting means it captures perceived reintegration, which may differ from objective social participation measures. Future studies should adopt longitudinal designs and include a broader set of variables to refine these findings.

### Future directions

This study underscores the need for personalized, multidimensional rehabilitation approaches tailored to each patient’s functional status. For patients with severe impairments, priority should be given to environmental adaptation and caregiver system improvement. For those with higher function, cognitive-linguistic rehabilitation, community mobility support, and socioeconomic reintegration should be emphasized. Longitudinal research is warranted to evaluate the long-term effects of such interventions and to develop functional-level-based rehabilitation models grounded in clinical evidence.
